# Predictors and Moderators of Internet- and Group-Based Cognitive Behaviour Therapy for Panic Disorder

**DOI:** 10.1371/journal.pone.0079024

**Published:** 2013-11-07

**Authors:** Samir El Alaoui, Erik Hedman, Brjánn Ljótsson, Jan Bergström, Erik Andersson, Christian Rück, Gerhard Andersson, Nils Lindefors

**Affiliations:** 1 Karolinska Institutet, Department of Clinical Neuroscience, Division of Psychiatry, Stockholm, Sweden; 2 Karolinska Institutet, Department of Clinical Neuroscience, Division of Psychology, Stockholm, Sweden; 3 Karolinska Institutet, Department of Clinical Neuroscience, Osher Center for Integrative Medicine, Stockholm, Sweden; 4 Stockholm University, Department of Psychology, Stockholm, Sweden; 5 Linköping University, Department of Behavioural Sciences and Learning, Linköping, Sweden; Bellvitge Biomedical Research Institute-IDIBELL, Spain

## Abstract

Internet-based cognitive behaviour therapy (ICBT) can be equally effective as traditional face-to-face cognitive behaviour therapy (CBT) for treating panic disorder (PD). However, little is known about the predictors and moderators of outcome of ICBT when delivered in psychiatric outpatient settings. This study investigated a selection of outcome predictors and moderators of ICBT for panic disorder based on data from a randomised controlled trial where therapist-guided ICBT was compared with group CBT (GCBT) for panic disorder. Participants (N = 104) received 10 weeks of ICBT or GCBT and were assessed before and after treatment, and after six months. Multiple regression analyses were used to test for significant predictors of treatment outcome. Predictors of positive treatment response for both modalities were having low levels of symptom severity and work impairment. In addition, anxiety sensitivity was found to have a small negative relationship with treatment outcome, suggesting that anxiety sensitivity may slightly enhance treatment response. Treatment modality had a moderating effect on the relationship between domestic impairment and outcome and on the relationship between initial age of onset of panic symptoms and treatment outcome, favouring ICBT for patients having had an early onset of PD symptoms and for patients having a high domestic functional impairment. These results suggest that both ICBT and GCBT are effective treatment modalities for PD and that it is possible to predict a significant proportion of the long-term outcome variance based on clinical variables.

## Introduction

Cognitive behaviour therapy (CBT) is effective in treating panic disorder (PD) [1]–[3] and internet-based CBT (ICBT) with guided therapist contact has received empirical support as an alternative treatment delivery format [4]–[7]. Our research group has previously evaluated the effectiveness of ICBT for PD in a regular psychiatric setting where ICBT was compared with group CBT (GCBT) in a randomised controlled trial [8]. Treatment significantly improved symptom severity in both groups with within-group effect sizes of *d* = 1.73 for ICBT and *d* = 1.63 for GCBT and no significant difference between groups. These results were sustained at 6-months follow-up. However, as some patients do not respond to treatment, there is a need to supplement existing evidence from ICBT studies with investigations of outcome predictors and moderators.

As for conventional (face-to-face) CBT for anxiety disorders, findings from previous research on prognostic factors are inconsistent across different psychiatric disorders [9]–[13]. For the treatment of PD specifically, demographic variables appear to lack consistent associations with treatment outcome [14]–[17] while there is indication that co-morbid psychiatric diagnoses may have a negative impact on outcome [18], [19], including agoraphobia [18], [20]–[27], depression [14], [25], [28], [29], generalised anxiety disorder [24] and personality disorders [14], [18], [26]. In a study by Dow et al [17] several significant outcome predictors of CBT for PD were identified, including agoraphobic avoidance, age of initial onset of panic symptoms, presence of co-morbid social phobia and fear of blood or injury. Sharp and Power [25] have also found a negative relationship between agoraphobic avoidance and treatment outcome, and Haby et al [30] found in a meta-regression study that severity of panic symptoms was negatively associated with treatment effect. Anxiety sensitivity has also been found to affect panic symptomatology. For example, Benítez et al. [31] reported that anxiety sensitivity had a significant effect on the course of PD episodes in a linear regression model associating ASI total score with length of PD episode (β = 0.77, *t* = 3.22, *p* = 0.0016). Also, in a study of treatment for PD by Meuret et al. [32] it was found that symptom appraisal (i.e. “fear of anxiety”) had an effect on panic symptoms.

In regard to ICBT as treatment modality for PD there is little published information specifically examining outcome predictors and moderators. In a study where ICBT was compared with face-to-face CBT, agoraphobic avoidance predicted poorer response in face to face CBT but not in ICBT and self-reported symptoms of personality disorder traits within the anxious cluster was related to negative treatment outcome in the ICBT condition, but was associated with a positive outcome in the face-to-face treatment condition [33].

Consequently, there is limited knowledge whether individual characteristics are differentially related to outcome in the treatment of PD when ICBT is compared with face-to-face CBT. A further investigation of which factors are likely to predict treatment effectiveness is valuable for a number of reasons. First, identifying variables related to outcome across different treatment modalities will allow better prediction of those who may benefit from face-to-face or ICBT; second, it would lead to a better understanding of variables that influence treatment mechanisms; and third, it could improve tailoring of treatment strategies to individual patient’s needs [34].

As this study was based on a large-scale trial in which ICBT was compared to GCBT in regular care, we regarded this as a very suitable cohort in which to investigate outcome predictors of CBT for PD. As the content of the two treatments was similar (with the exception of delivery format and mode of therapist contact) these data provided an excellent opportunity for investigating whether patient characteristics influence the outcome differently depending on treatment modality. Consequently, the aim of the present study was to investigate outcome predictors and moderators of ICBT and GCBT for panic disorder based on data from that trial [8]. Based on outcome predictors identified in the studies above, we were interested in examining their possible relationship with treatment outcome in our ICBT vs. GCBT trial data. However, since the analyses in our study are based on data from an earlier effectiveness trial, the numbers of available predictors were limited to the data collected in that study. Consequently, we were able to test the following clinical variables for their effects on treatment outcome: symptom severity, age of onset, duration of illness, anxiety sensitivity and the presence of co-morbid agoraphobia. Additionally, we would test the effect of available demographic variables, namely age, gender, employment status and sick leave as well as variables related to the therapeutic process (e.g. treatment adherence). In addition, data on functional impairment were collected during the trial, and we were interested in examining whether this might have an effect on treatment response.

As previous findings on outcome predictors and moderators have been heterogeneous, the analyses were conducted mainly from an exploratory viewpoint, looking at how treatment response might be associated with the selection of pre-treatment factors discussed above.

## Methods

### Study Design

Data (N = 104) from a previous trial [8] (ClinicalTrials.gov identifier: NCT00845260) investigating the effect of ICBT and GCBT were used to analyse potential predictors and moderators. A detailed account of the outcome study is reported elsewhere [8]. Patient level data are archived at Karolinska institutet/Stockholm county council and are available upon request in an unidentifiable format.

### Sample and Recruitment

**Figure 1:**
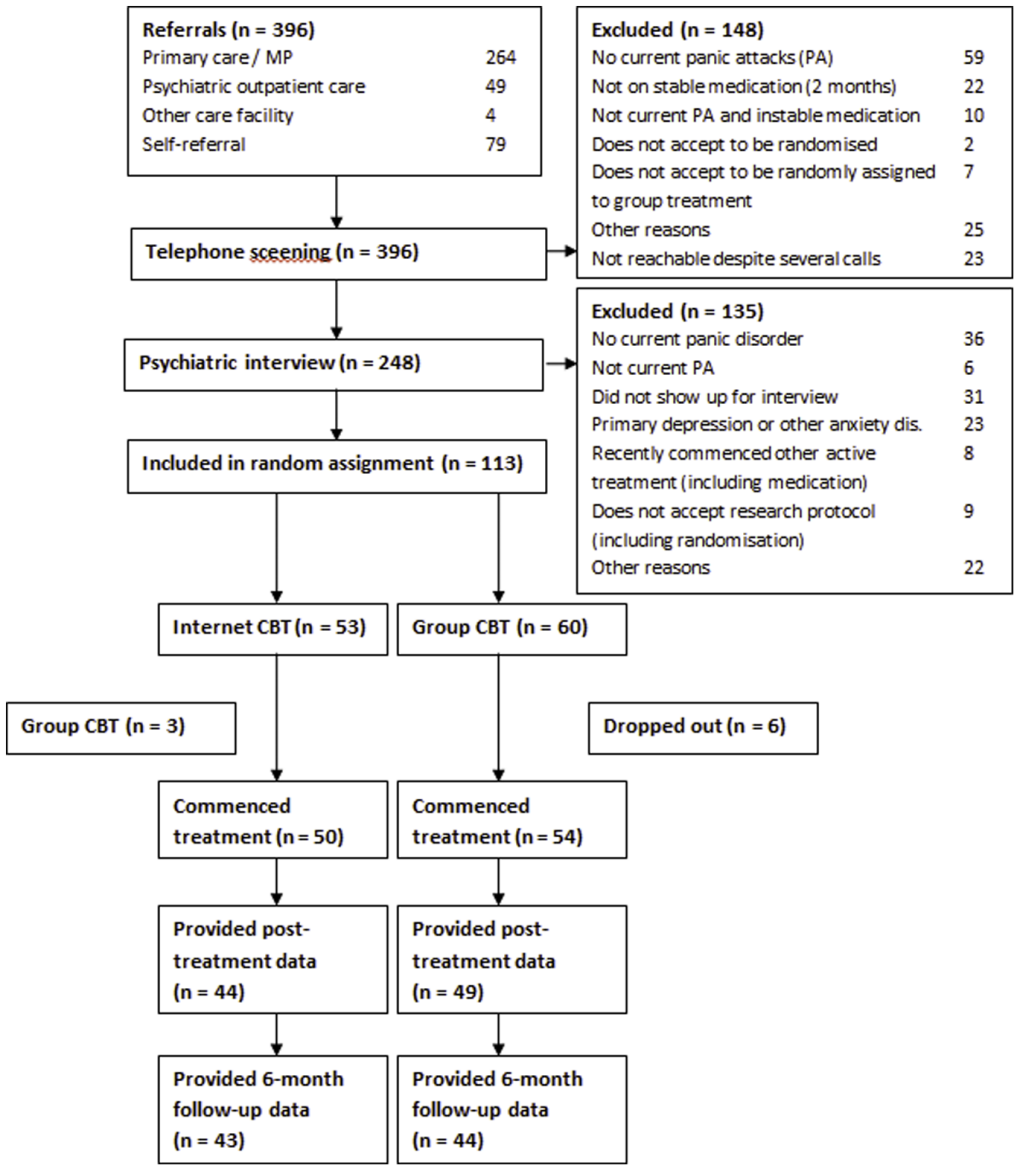
Flowchart of study participants, point of random assignment, and dropouts.

Of 396 referrals, 148 participants were excluded after a telephone-screening interview, and an additional 135 were excluded after psychiatric interviews. The remaining 113 participants were randomised to either ICBT or GCBT. After a dropout of nine participants, 50 participants started ICBT and 54 started GCBT. Figure 1 presents a flowchart of the participant flow [8]. The characteristics of the sample regarding clinical and demographic variables are presented in Table 1.

**Table 1:** Demographic and clinical characteristics of the participants.

			Treatment group			
	Total (n = 104)		ICBT (n = 50)		GCBT (n = 54)	
Age, years: mean (SD)	34.2	(9.4)	33.8	(9.7)	34.6	(9.2)
Gender: female, %	62%		64%		59%	
Age of initial onset of panic symptoms, mean (SD)	25.6	(9.1)	25.6	(8.9)	25.6	(9.4)
Duration of illness (PD), mean years (SD)	6.7	(9.4)	6.0	(9.3)	7.3	(9.6)
Severity of panic disorder (PDSS), mean (SD)	14.2	(4.1)	14.1	(4.3)	14.2	(4.0)
Functional impairment in work/school (SDS1), mean (SD)	5.7	(3.1)	5.5	(3.3)	5.9	(2.9)
Functional impairment in social life (SDS2),mean (SD)	5.8	(2.6)	5.6	(2.8)	5.9	(2.5)
Functional impairment in family life/home responsibilities (SDS3), mean (SD)	4.4	(2.7)	4.1	(2.9)	4.6	(2.5)
Impaired Performance Days (WQ2), mean (SD)	4.7	(6.4)	3.4	(5.0)	5.8	(7.3)
Number of sick-leave days (WQ1), mean (SD)	5.5	(8.1)	5.5	(8.3)	5.6	(7.9)
Anxiety Sensitivity Index (ASI), mean (SD)	32.9	(12.0)	32.5	(11.6)	33.2	(12.4)
Psychotropic medication, %	45%		44%		46%	
Agoraphobia, %	85%		86%		83%	
Any anxiety disorder, %	17%		16%		18%	
Depression, %	10%		8%		11%	
Generalised anxiety disorder, %	11%		12%		9%	
Health anxiety (hypochondriasis), %	3%		0%		6%	
Social anxiety disorder, %	6%		4%		7%	
Specific phobia, %	2%		0%		4%	
Obsessive compulsive disorder, %	1%		0%		2%	
Posttraumatic stress disorder, %	1%		0%		2%	

Abbreviations: ICBT, Internet-based cognitive behaviour therapy; GCBT, group-based cognitive behaviour therapy; WQ, work questionnaire; ASI, Anxiety Sensitivity Index; SDS, Sheehan Disability Scale.

Participants were recruited by referrals from either psychiatric outpatient clinics or general practitioners. Approximately one-third of participants were self-referred to the psychiatric clinic at the Karolinska University Hospital in Stockholm, Sweden, where the trial was conducted. Psychiatrists conducted diagnostic interviews based on the following inclusion criteria: a) participants had to fulfil the DSM-IV criteria for PD (with or without agoraphobia), according to the Mini-International Neuropsychiatric Interview (MINI) [35]; b) PD had to be the primary diagnosis; c) participants had to be over 18 years of age; d) no presence of co-morbid severe depression or suicidal ideation; e) dose of medication held constant for 2 months prior to the study, if on prescribed medication for PD; and, f) no other concurrent psychological treatment during the study. A more detailed description of the inclusion criteria and the recruitment procedure is available in the original report [8].

The study protocol from the original RCT was approved by the Regional Ethical Review Board, Stockholm, Sweden. Written informed consent was obtained from all participants after the procedure had been fully explained by the psychiatrists.

### Treatment Interventions

Participants were randomly assigned to 10 weeks of either ICBT or GCBT. The content of the treatments was based on established CBT principles [36]. In both conditions, the treatment content was divided into 10 modules. The content of the modules was: 1) psycho-education; 2–3) cognitive restructuring; 4–5) interoceptive exposure; 6–9) in vivo exposure for agoraphobic situations; and 10) relapse prevention. Participants in both treatment conditions received identical self-help texts covering the topics of each module.

ICBT participants accessed the treatment through a password-protected website through which they were able to communicate with their assigned therapist. They were granted gradual access to the modules, contingent on completing homework exercises which they reported to their therapist through the online treatment platform. For three predefined workdays per week, the therapists were accessible on the ICBT website to provide regular written feedback on homework-related issues and other questions that might arise during treatment. Participants also had the possibility of anonymously discussing thoughts about their treatment with other participants in an online forum.

In the GCBT condition, participants received the treatment modules during weekly two-hour group sessions, with 5–6 participants in each group, supervised by two licensed clinical psychologists. In the group sessions, the psychologist explained the contents of each module and reviewed the participants’ homework exercises.

### Main Outcome Variables

#### Primary outcome measure

The primary outcome measure was the Swedish version of the clinician administrated Panic Disorder Severity Scale (PDSS; [37]) developed by Shear et al in 1997. This scale measures frequency of panic symptoms and attacks during the last month and the distress and worry participants report in relation to these attacks, level of social and work-related functional impairments, and avoidance behaviours. Test-retest reliability for the American version has been reported to be 0.71 with a Cronbach’s alpha of 0.88 [38]. However, we have found no psychometric data on the Swedish version of this scale.

#### Diagnostic assessment

As described above, the Mini-International Neuropsychiatric Interview (MINI) [35] was used to establish psychiatric diagnoses according to the Diagnostic and Statistical Manual of Mental Disorders (DSM-IV-TR) [39].

### Potential Predictors and Moderators

#### Demographic information

Demographic information was collected during diagnostic assessments within the clinical trial [8]. Variables included in the analyses of outcome predictors were age, gender, employment status, number of sick-leave days, and number of impaired performance days during the last 30 days.

#### Clinical characteristics

Data on severity of illness (measured by the PDSS), age of onset, duration of illness, presence of co-morbid disorders, and concurrent use of stabilised psychotropic medication were collected through standardised interviews performed by a psychiatrist on each measurement occasion using the MINI as diagnostic instrument. Further, the Anxiety Sensitivity Index (ASI) [40] was used to measure fear of anxiety-related symptoms. The ASI consists of 16 items with 5-point scales of scores ranging between 0 and 4. The range of the total score is 0–64. It’s reliability and validity has been documented by a vast number of peer-reviewed studies [41]. The Montgomery Åsberg Depression Rating Scale (MADRS) [42] was used to measure depressive symptoms and consists of ten items with each ranging in scores between 0–6. The total score ranges between 0 to 54. Total score ranges between 0–60. The instrument has a high reliability [42] and also demonstrates high correlations between expert and self-rating versions of the MADRS (from *r* = 0.80 to 0.94) [43]. Functional impairment was evaluated with the Sheehan Disability Scale (SDS) [44] to assess disabilities in three related domains: work/school activities, social functioning, and family relationships/home responsibilities. The SDS consists of a 10-point visual analogue scale with numeric, verbal description, and spatio-visual anchors for assessing disability in each domain.

#### Therapy process-related measures

In the outcome study [8], patient compliance to treatment was defined as having completed at least five modules in the ICBT condition or at least five group sessions in the GCBT condition. The argument for defining five modules/sessions as a meaningful cut-off for compliance is that the central components of the treatment would be completed by this stage, including psycho-education, cognitive restructuring, and exposure to feared stimuli. 37 of 50 (74%) participants in the ICBT group completed at least five modules and 51 of 54 (94%) participants completed at least five sessions in the GCBT group. In the present study, treatment adherence was considered as a potential predictor variable.

### Procedure

Outcome data were collected at baseline, post-treatment, and at six-month follow-up. During these occasions, the psychiatrists performing the clinical interviews were blind to treatment conditions. Further details of the procedure are described in the original paper [8].

### Data Analysis

Statistical analyses were performed using SPSS version 20 [45].

### Missing Data

Since there were some missing data due to attrition at post-treatment (88% provided post data in the ICBT condition and 91% in the GCBT condition) and at follow-up (86% provided follow-up data in the ICBT condition and 82% in the GCBT condition), we performed a parallel analysis on a second data set in order to compare the possible effect of missing data on the final results. Missing values were imputed using multiple imputation of missing values. The Missing Value Analysis module was used with the EM method in SPSS [46]. Little’s MCAR test confirmed that data in the original data set were missing completely at random (Chi-Square = 36.655, *DF* = 3191, *p* = 1.000). The results from these parallel analyses on the imputed data set did not differ from the analysis with the completers’ only dataset; therefore, we decided to use only the original dataset (i.e. using no imputed data) during the analyses presented in this paper.

### Regression Analyses

If there is a significant relation between an independent variable and the outcome variable, the independent variable could be said to be a predictor of the outcome [47]. For example, if there is a relationship between the duration of illness and treatment outcome, the number of years that patients have had panic disorder might predict how well these patients are likely to respond to treatment. Further, the strength of this relationship might be explained by a third variable. This variable is then said to moderate the relationship. For example, treatment condition might influence the relation between duration of illness and treatment outcome in that this relation might be stronger in one treatment modality (such as ICBT) as compared to another treatment modality (such as GCBT). More formally described, in an RCT a moderator is a pre-randomization variable that has an interactive effect with treatment on outcome [34].

In order to investigate potential predictor variables, exploratory regression analyses of demographic data and clinical data were performed using hierarchical linear regression procedures. For increased interpretability of interactions, and in accordance with recommendations by Aiken and West [48], continuous predictor variables were grand-mean centred prior to analysis. This was done by subtracting the grand-mean of a variable from each of its individual values. To be able to compare effects of predictors for each treatment group, treatment condition was coded using a dummy variable, where 0 = GCBT and 1 = ICBT. This allowed us to explore how the effects differ between treatment modalities. As recommended by Jaccard and Turrisi [49] dummy variables were not centred.

A four-predictor regression equation was used as a model for outcome prediction from a set of independent variables (including the PDSS baseline control variable) with a product term (*Y * =  α + b_1_ *Q* + b_2_ *X* + b_3_ *Z* + b_4_ *XZ* + ε) where, *Q, X* and *Z* stand for predictor variables, and the product term *XZ* stands for the moderating interaction variable. The strategy proposed by Cohen and Cohen [50] is a common approach for modelling interaction effects in multiple regression. The interaction effect is calculated by forming the product term *XZ* from the two independent variables of interest. In the present study, the interaction term is represented by the product of the GROUP variable and the target predictor variable (i.e. a non-PDSS baseline variable), for example ASI×GROUP. This product term allows testing for the existence of a moderated relationship. Initial symptom severity was controlled for in all regression analyses and was included as an independent variable. Single variables were centered in the first block, and two-way interaction terms in the second block. Thus, two R^2^ values were calculated, one value for the model containing only the main effects, and then one value for the model also containing the product term PREDICTOR×GROUP. If the difference between the R^2^ values of the two models was statistically significant, an interaction effect was considered present. Independent variables with a significant main effect, but no interaction effect, were considered as predictor variables [49].

**Figure 2:**
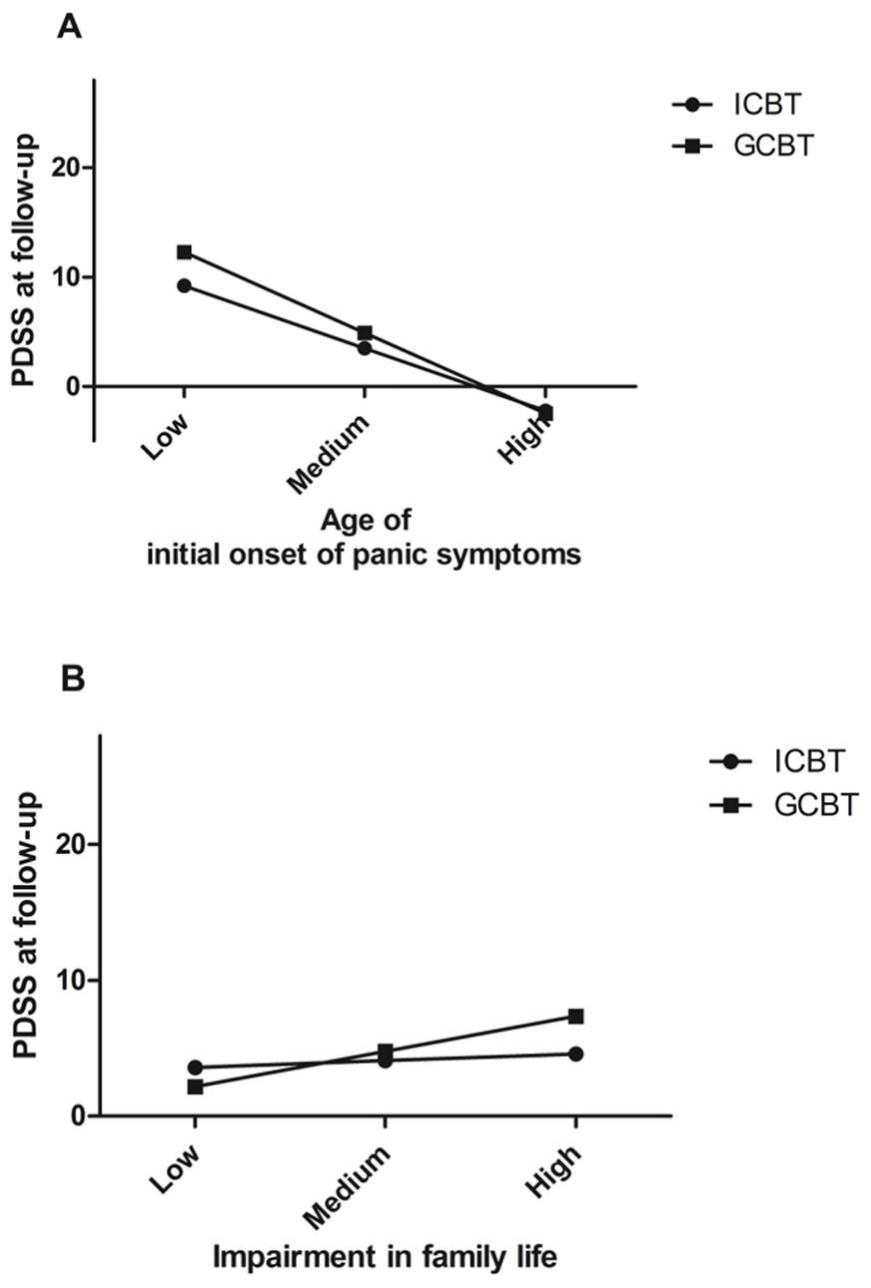
Representation of the observed moderator effects of treatment modality. The top graph (A) illustrates the observed moderator effect of treatment modality on the initial age of onset of panic symptoms - treatment outcome relationship. The bottom graph (B) illustrates the observed moderator effect of treatment modality on the functional impairment in family life - treatment outcome relationship. Functional impairment in family life is based on the third subscale of the Sheehan Disability Scale. The main effects (i.e. initial age of onset of panic symptoms and domestic impairment) are displayed on the x-axis, the dependent variable (PDSS) is displayed on the y-axis, and the moderating variable (treatment modality) is represented by the two separate lines. Three levels (low, medium and high) were calculated using the mean as the medium value, one standard deviation below the mean as the low mean and one standard deviation above the mean as the high mean. Mean age of onset was 25.6 years. with a standard deviation of 9.1. Mean score on functional impairment in family life was 4.4 with a standard deviation of 2.7. Abbreviations: PDSS, Panic Disorder Severity Scale; ICBT, internet-based cognitive behavioural treatment; GCBT, group-delivered cognitive behavioural treatment. Note: negative values on the y-axis are possible as values in the figure are based on estimated parameters.

Predictors and moderators from the initial regression models with beta estimates of *p*<0.10 were included in a final model by backward/forward deletion in order to obtain a model where each included predictor/moderator made an additional contribution to explaining the variance in the outcome variable. For exploratory purposes, significant two-way interactions were further analysed in a three-way interaction model. As recommended by Cohen and Cohen [50], interactions were graphed in order to facilitate interpretation. ModGraph-I [51]was used to compute cell means for the graphical display of moderator analyses presented in Figure 2.

## Results

### Attrition

Out of 50 participants who started treatment in the ICBT condition, 44 (88%) provided post-treatment data, and 43 (86%) provided follow-up data. Out of 54 participants who were treated in the GCBT group, 49 (91%) provided post-treatment data and 44 (82%) provided follow-up data.

### Adherence

At post-treatment, 88 (85%) of 104 participants had complied with treatment (i.e. having completed at least five modules or group sessions). Association between adherence and PDSS both at post-treatment and follow-up was non-significant.

### Treatment Effectiveness

The standardised within-group effect size between pre-treatment and 6-month follow-up was *d* = 2.35 (Hedges’ *g* 2.33, 95% CI: 1.43–3.23) for the ICBT group, and *d* = 1.92 (Hedges’ *g* 1.9, 95% CI: 0.89–2.91) for the GCBT condition, with a standardised between-group effect size of *d* = 0.23 (95% CI: −0.15–0.62).

### Predictors of Treatment Outcome

Several statistically significant predictor variables were identified in the initial regression analyses, namely the number of sick leave days reported prior to treatment (WQ1), pre-treatment functional impairment in work/school (SDS1), pre-treatment functional impairment in family relationships/home responsibilities (SDS3) and pre-treatment level of anxiety sensitivity (ASI). The results from these analyses are presented in Table 2. Also, baseline PDSS scores predicted PDSS scores at six-month follow-up (see Table 3).

**Table 2:** Initial significant models with PDSS scores at six-month follow-up as dependent variable.

	Predictor					Predictor×Group				
Variable atpre-treatment	B	S.E.	β	t	*p* value	B	S.E.	β	t	*p* value
SDS1_c_	0.711	0.243	0.453	2.920	0.005	−0.437	0.300	−0.212	−1.455	0.149
SDS3_c_	0.637	0.291	0.362	2.193	0.031	−0.885	0.341	0.384	−2.592	0.011
ASI_c_	−0.003	0.054	−0.007	−0.047	0.962	−0.154	0.075	−0.270	−2.042	0.044
WQ1_c_	0.197	0.095	0.320	2.075	0.041	−0.021	0.119	−0.260	−0.180	0.858

Abbreviations: SDS1_c_, grand-mean centred score of the work/school domain of the Sheehan Disability Scale; SDS3_c_, grand-mean centred score of the family relationships/home responsibilities domain of the Sheehan Disability Scale; ASI_ c_, grand-mean centred score of the Anxiety Sensitivity Index; WQ1_c_, number of sick-leave days.

**Table 3:** Multivariate linear regression presenting the final model with PDSS scores at six-month follow-up as dependent variable.

	B	SE	β	*p* value
*Model*				
*R* = .667		3.92		<0.05
*R^2^* = .445				
Adj *R^2^* = .402				
*Predictor variables*				
SEVERITY	0.5	0.13	0.42	<0.001
ASI	−0.09	0.04	−0.23	<0.05
SDS1	0.49	0.17	0.30	<0.01
*Moderators*				
SDS3×GROUP	−1.10	0.35	−0.30	<0.01
ONSET×GROUP	0.17	0.78	0.21	<0.05

Abbreviations: SEVERITY, Baseline symptom severity measured with the Panic Disorder Severity Scale; ASI, Anxiety sensitivity Index; SDS1, Functional impairment in work/school; SDS3, Functional impairment in family life/home responsibilities; ONSET, Age of initial onset of panic symptoms; GROUP, treatment condition, i.e. internet-delivered cognitive behavioural therapy or group-administered cognitive behavioural therapy.

Sick leave from work and reported functional impairment in work or school are two variables that reflect work disability. Regression analysis reported a significant main effect (β = 0.32, *p*<0.05) for sick leave days, suggesting that there was a positive association between the number of sick leave days and treatment outcome after initial symptom severity had been controlled for. The second indicator of work disability identified as being associated with treatment outcome was functional impairment in the work/school domain (SDS1). Similar to the effect of sick leave days, regression analysis demonstrated a main effect (β = 0.5, *p*<0.001) of SDS1, suggesting that functional impairment may be positively associated with outcome symptom severity in both treatment modalities after initial PDSS scores has been controlled for.

The third identified prognostic variable was domestic impairment (SDS3) which was found to have a significant main effect on treatment outcome (see Table 2).

### Moderators of Treatment Outcome

In addition, an interaction effect was identified in the initial analyses for baseline anxiety sensitivity (ASI; β = −0.3, *p*<0.05), suggesting that treatment modality may moderate the relationship between ASI and PDSS outcome. According to this model, panic symptoms at six-month follow-up did not differ between the two treatment conditions under conditions of low anxiety sensitivity, but there was a statistically significant difference under conditions of high anxiety sensitivity. However, this interaction effect was not significant in the final regression model. In the final model (see Table 3), predictors and moderators accounted for approximately 45% of the outcome variance. Baseline PDSS scores, ASI and SDS1 were identified as predictor variables, and two relationships were identified as being moderated by treatment modality, namely the relation between SDS3 and outcome PDSS at follow-up and the relation between age of onset of panic symptoms and outcome PDSS at follow-up.

The first relationship identified as being moderated by treatment modality in the final regression model was participants’ age of initial onset of panic symptoms. The total sample mean (SD) age of onset was 25.6 (9.1). According to the results from this regression model, panic symptoms at six-month follow-up differed between treatment modalities for participants having had a late onset of PD symptoms. Figure 2 (A) illustrates a representation of the observed moderator effects of treatment modality on the initial age of onset of PD symptoms - treatment outcome relationship, where “medium” refers to the sample mean age (i.e. 25.6 years.), “low” refers to one standard deviation below the mean (i.e. 16.5 years.) and “high” refers to one standard deviation above the mean (i.e. 34.7 years.).

Domestic impairment (SDS3) was, in addition to its main effect, found to have an interaction effect (see Table 2) where ICBT displayed the largest improvement (a 13.4 point decrease in PDSS for the ICBT condition between pre-treatment and follow-up, versus a 7.4 point decrease for the GCBT condition). Figure 2 (B) illustrates the relationship between outcome PDSS scores and treatment modality for different levels of domestic impairment.

Finally, significant two-way interaction effects (ASI and SDS3) from the initial regression analyses were computed in a three-way interaction model. The results suggested that anxiety sensitivity was only predictive for individuals with a high degree of functional impairment receiving ICBT, although slightly above the conventional alpha level of 0.05 (β = -0.278, *R* ^2^ = 0.369, *p* = 0.07).

## Discussion

As there is little information about the variables affecting the treatment response of ICBT for PD delivered in regular care settings and how it compares to traditional group CBT, this study was mainly exploratory with the aim of analysing a set of candidate predictor variables. As mentioned in the introduction, there appears to be few stable predictors across studies and diagnoses. However, we were able to replicate a few of these and the final regression model explained about half of the outcome variance.

## Results

The final regression model identified several outcome predictors. In line with previous research (see for example the study by Dow et al [17]), we were able to identify the association between baseline panic symptom severity (i.e. pre-treatment PDSS scores) and outcome level of panic symptoms (i.e. six-month follow-up PDSS scores). We also replicated the association between age of initial onset of panic symptoms with treatment outcome, where patients having had an early onset of panic symptoms tends to have poorer outcome compared to patients having had a later onset of panic symptoms.

Anxiety sensitivity was also identified as an outcome predictor. Interestingly, in the initial regression analyses on the role of anxiety sensitivity, a moderating effect was also found for treatment modality. A small but significant negative relationship between anxiety sensitivity and PDSS outcome was found in the ICBT condition, where participants having higher levels of anxiety sensitivity seemed to respond more favourably to treatment than participants having scored low on ASI. A possible explanation for this negative relationship might be due to the finding that patients scoring high on anxiety sensitivity tended to respond more to fearful stimuli (see for example the study by Holloway and McNally [52] on the effects of anxiety sensitivity on response to hyperventilation). Consequently, as fear presentation is likely to be a prerequisite for exposure, it is possible that having a high level of anxiety sensitivity leads to a greater habituation response to exposure of fearful stimuli since the intensity of exposure is likely to be elevated compared to individuals having a low level of anxiety sensitivity.

Another area of observation concerned the relation between occupational functioning and treatment response. Normal functioning at work, including having few sick leave days, appeared to play a significant role in how individuals responded to treatment, regardless of treatment modality. These results were in accordance with a previous study on predictors of ICBT for SAD [12], where employment status (i.e. working full-time) predicted a positive outcome, irrespective of treatment format.

Treatment modality was found to have a small but statistically significant moderating effect on two relationships. First, although domestic impairment was associated with poorer treatment outcome (also in line with the study by Dow et al [17]) participants who were experiencing greater functional impairment in the family life/home responsibilities domain seemed to benefit more from treatment in the ICBT modality. Second, the results from the moderator analysis suggest that patients with an early onset might benefit slightly more from ICBT than from GCBT. However, since the mean differences between the two treatments modalities in outcome PDSSS scores are only approximately 2 points, this moderating effect might not be clinically relevant; CBT treatment is after all effective in both modalities.

Finally, there were findings we were not able to replicate. For example, we found no significant association between co-morbid disorders and treatment response, which stands in contrast to other studies where depression [14], [25], [28], [29] and generalised anxiety disorder [24] was found to have an effect on treatment response.

### Clinical Implications

Although a number of statistically significant outcome predictors and moderators were identified, a discussion on the clinical implications of these is warranted. For example, should the decision to include or exclude patients seeking care be based on screening scores of Sheehan Disability Scale or recent amount of sick leave days? It seems that, on average, treatment is effective regardless of baseline values on these variables. However, the level of post-treatment symptom severity generally differs among patients depending on their initial symptom levels.

### Limitations

Several limitations of this study need to be considered. First, the lack of an untreated control group made it difficult to control for spontaneous recovery, although that was not the focus of the present study. However, the high exclusion rate for the trial, combined with the limited sample size, may represent a threat to the ecological validity.

Second, due to a limited sample size, statistically significant interaction effects might have been difficult to establish.

Third, a general concern due to the inherent restrictions of an RCT study design is the possible exclusion of patients with characteristics corresponding to the most salient and clinically relevant predictors and moderators of outcome. This implies that the results may have limited generalizability due to the restrictive inclusion and exclusion criteria of RCTs (compared to, for example, observational studies). Some authors caution that the treatment effects of RCT might be overstated in relation to the general population receiving the same treatment, which could be explained by the increased likelihood of treatment adherence of patience in RCT studies [53].

### Conclusions

Despite the limitations discussed above, this study provides further knowledge about patient characteristics that may have an impact on differences in panic disorder symptom severity at six-month follow up depending on treatment modality for patients with PD who seek treatment in psychiatric care. Specifically, panic symptom severity and work disability was found to predict levels of panic symptoms after treatment, and anxiety sensitivity seems to have a small effect on outcome in that anxiety sensitivity might slightly enhance the treatment effect. Further, treatment modality seems to have a moderating effect on two relationships, namely the domestic impairment - treatment outcome relationship and the initial age of onset of panic symptoms - treatment outcome relationship, favouring the ICBT format for patients having had a late onset of PD symptoms and for patients having had a high level of functional impairment in the family/home responsibilities domain.

Although these findings may not warrant new suggestions on selection criteria for treatment or modifications to treatment protocols, clinicians may benefit from an increased awareness of how baseline differences in patients seeking psychiatric care for PD can have an impact on treatment response. The results of this study pose some additional suggestions for future research on ICBT; for example, the investigation of the therapist’s role in guiding exposure assignments in ICBT and determining how the method of therapist guidance might be studied as a potential mediator of treatment effectiveness.
